# Development of a core outcome set for hypertensive intracerebral hemorrhage in clinical trials of traditional Chinese medicine: a study protocol

**DOI:** 10.1186/s13063-022-06801-z

**Published:** 2022-10-12

**Authors:** Min Jia, Yan Lu, Xiao Liang, Chenguang Tong, Jian Wang, Jun Tang, Jian Yang, Min Wang, Weiwei Jiao, Wanqing Du, Jingjing Wei, Zixiu Zeng, Zhenmin Xu, Qian Chen, Lin Lei, Xing Liao, Yunling Zhang

**Affiliations:** 1 Xiyuan Hospital, China Academy of Chinese Medical Sciences, Beijing, China; 2 Center for Evidence-based Chinese Medicine, Institute of Basic Research in Clinical Medicine, China Academy of Chinese Medical Sciences, Beijing, China; 3 The Affiliated Hospital to Changchun University of Chinese Medicine, Changchun, China; 4 Chongqing Traditional Chinese Medicine Hospital, Chongqing, China; 5 Graduate School, Beijing University of Chinese Medicine, Beijing, China

**Keywords:** Core outcome set, Hypertensive intracerebral hemorrhage, Clinical trials, Traditional Chinese medicine, Protocol

## Abstract

**Background:**

Intracerebral hemorrhage (ICH) is a devastating disease, its mortality and disability rate are high. In China, hypertensive intracerebral hemorrhage (HICH) is responsible for 75% of all the cases of primary ICH. A lot of randomized controlled trials (RCTs) of traditional Chinese medicine (TCM) for treating HICH have been carried out. However, these RCTs have a lot of problems, such as heterogeneous outcomes, non-uniform point of measurement. These lead to systematic review/meta-analysis only can include a small number of studies. And outcome measures did not take the wishes of patients and other stakeholders into account. The aim of this study is to establish the core outcome set (COS) for future TCM clinical trials of HICH.

**Methods and analysis:**

First, we will develop a long list of general outcomes by making systematic literature review and semi-structured interviews. Then healthcare professionals and patients with HICH will be invited to participate in two rounds of the Delphi survey to determine the importance of the outcome. Finally, a face-to-face consensus meeting will be conducted to determine the final COS of HICH, including what outcomes should be measured and when and how to measure the outcomes.

**Results:**

We aim to develop a COS that includes TCM core syndrome for HICH to determine what outcomes should be reported and when and how to measure them.

**Conclusion:**

By doing this, we can increase the reporting consistency and reduce the reporting bias in the outcome, which leads to the reuse of research data in meta-analysis and the making of informed healthcare decisions.

**Ethics and dissemination:**

The entire project has received approval from the Ethics Committee of Xiyuan Hospital, China Academy of Chinese Medical Sciences. The final COS will be published and reported at the national and international conferences.

**Trial registration:**

This study is registered with the Core Outcome Measures in Effectiveness Trials database as study 1475. Registered on December 2019.

## Strengths and limitations of this study

1. To make comprehensive literature retrieval, in addition to the four Chinese databases and four English databases, two trial registries will also be searched.
2. Health professionals participating in the Delphi survey and consensus meeting come from different regions, which is conducive to reducing the impact of regional differences.
3. Different stakeholders will be invited to the development of the COS, to ensure that the wishes of patients and other stakeholders will be taken into account.
4. When and how to measure the included outcomes will be recommended at the consensus meeting.
5. Patients will be recruited only in Xiyuan Hospital, China Academy of Chinese Medical Sciences.

## Introduction

Intracerebral hemorrhage (ICH) is a devastating disease, which refers to non-traumatic intracerebral parenchymal hemorrhage, whose main symptoms are headache, nausea, vomiting, and neurological deterioration [1]. More than 2 million people suffer from this disease each year globally, accounting for about 10–15% of all strokes [2, 3]. Although its morbidity is lower than that of cerebral infarction, its mortality is higher. Some research finds that the mortality rates of within 30 days of onset and within one year of illness respectively were 37–52% and 54% [4]. Moreover, since it can lead to severe neurological impairment, it is also associated with high levels of disability. Fewer than 20% of survivors have long-term functional independence in the chronic stage of ICH at 6 months and 36% of survivors remaining moderately to severely disabled at discharge [3, 5]. Hypertension is the primary and strongest risk factor (odds ratio [OR], 9.18[95%CI,6.80-12.39]) for ICH [6, 7]. Hypertension can induce small, arterial perforator degenerative changes, which are thought to increase the likelihood of rupture. Hypertensive intracerebral hemorrhage (HICH) occurs mainly in deep brain structures, for example, basal ganglia, thalamus and brainstem, which are supplied by these degenerative arterial perforators [8]. In China, HICH is responsible for 75% of all the cases of primary ICH [9]. So it is a great challenge for researchers and clinicians to improve the treatment of hypertensive intracerebral hemorrhage and improve the prognosis of patients.

Current western medicine treatment mainly focuses on methods for rapid reduction in blood pressure, removal of the hematoma, management of secondary complications, such as control of intracranial pressure and reduction of cerebral edema, and supportive therapies, for example, management of glucose level, oxygenation, and circulation. But, currently, there are no effective pharmacologic or surgical therapies to improve the survival and functional recovery in patients with HICH [10, 11].

HICH belongs to the category of stroke (Zhong Feng) in traditional Chinese medicine (TCM). In China, based on the TCM theory of syndrome differentiation, herbal medicine, acupuncture, and other non-medication TCM therapies have been used to treat stroke for thousand of years [12, 13]. And, a lot of randomized controlled trials (RCTs) of TCM for treating HICH have been carried out to prove the curative effect of TCM.

Some studies indicate that TCM combined with western medicine therapy appears to be effective in patients with acute hypertensive ICH, for example improving neurological deficit scores and reducing the volume of hemorrhage [9, 14].

However, these RCTs have a lot of problems, such as heterogeneous outcome, fuzzy definition of outcome, non-uniform point of measurement, and lack of reporting primary or long-term outcomes [14]. These will lead to systematic review/meta-analysis only can include a small number of studies in quantitative synthesis, to remain homogeneous. It is not conducive to the secondary utilization of these research data. At the same time, outcome measures did not take into account the wishes of patients and other stakeholders. So it is urgent to establish the core outcome set (COS) of HICH.

## Rationale for the development of a COS

There is no defined COS for HICH. A COS is defined as the minimum outcomes that should be measured and reported in clinical trials in a specific area of healthcare [15]. By doing this, we can increase the reporting consistency and reduce the reporting bias in the outcome, which leads to the reuse of research data in meta-analysis and the making of informed healthcare decisions [16].

Treatment based on syndrome differentiation is a characteristic of TCM. So we will develop a COS that includes TCM core syndrome for HICH to determine what outcomes should be reported and when and how to measure them. First, a systematic literature review and qualitative interviews will be conducted to develop a long list of outcomes for HICH. Then, to determine the importance and priority of outcomes, two rounds of the Delphi survey with different stakeholders will be carried out. Finally, we will hold a consensus meeting to determine the COS. The COS will be published and used for future clinical trials to improve outcome reporting in HICH.

## Scope of the COS-TCM

We aim to identify the exact outcomes of patients with HICH that different stakeholders are concerned in RCTs of TCM, using a transparent methodology. At the same time, we intend to make a consensus on when and how these COS should be measured. The 13 minimum Core Outcome Set-STAndardised Protocol Items (the COS-STAP Statement) will be addressed in the protocol [17].

The scope of the COS-TCM is as follows:

1. Health condition: HICH.
2. Population: adults with HICH.
3. Interventions: TCM therapies, including traditional herbal medicine, acupuncture, moxibustion, cupping, massage, Qigong and other non-drug therapies.
4. Context of use: RCTs.

## Registration

This study is registered on the Core Outcome Measures in Effectiveness Trials database as study 1475 (available at: https://www.comet-initiative.org/Studies/Details/1475).

## Methods and analysis

### Stakeholders

In the COS development process, different stakeholders will be included, such as health professionals and patients. Health professionals will include TCM clinicians in cerebrovascular disease, Western medicine clinicians in cerebrovascular disease (neurologists and neurosurgeons), researchers interested in HICH, and methodologists in the field of evidence-based medicine. Clinician stakeholder eligibility is based on involvement in the clinical care of patients with HICH. Furthermore, authors who have published a significant volume of publications in the field of HICH will be invited to participate.

### Steering group

We will form a Steering group to oversee the development of the COS. The Steering group consists of five experts, such as two TCM experts, a neurosurgeon and neurologist in western medicine, and a methodologist in COS development. Their primary responsibility is to review and confirm research protocol, make decisions when in doubt, and participate in consensus meetings to promote the development of COS.

### Patient and public involvement

To get the outcome of patients concerned, patients or their representatives will be recruited to participate in the semi-structured interviews and two rounds of the Delphi survey.

### Design

We will develop this COS in three Phase:

In phase 1, by making a systematic literature review and semi-structured interviews, we will develop a long list of general outcomes.

In phase 2, we will carry out two rounds of the Delphi survey with different stakeholders.

In phase 3, a face-to-face consensus meeting will be conducted to determine the final COS of HICH, including what outcomes should be measured and when and how to measure the outcomes.

**Fig. 1:**
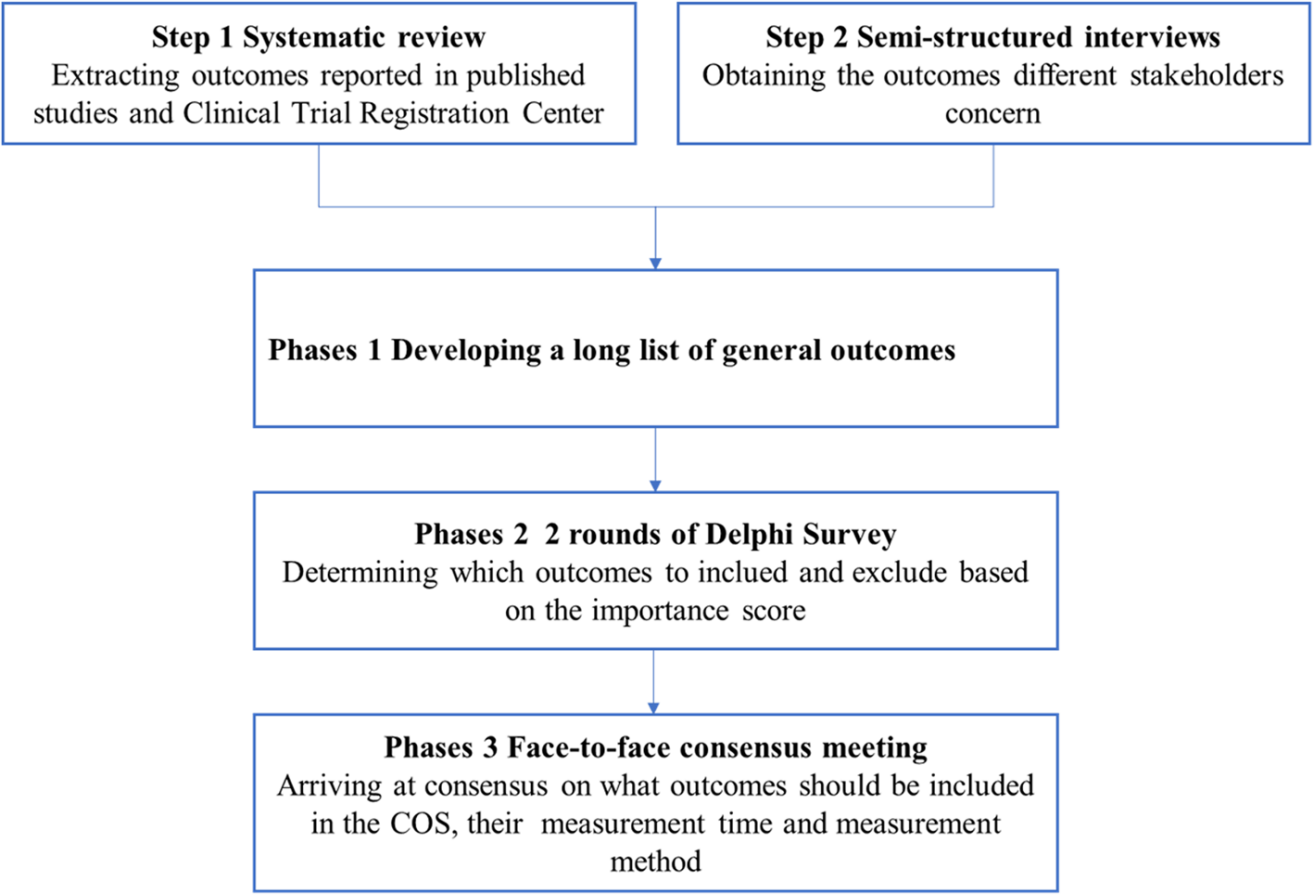
Flowchart for developing a COS for HICH in clinical trials of TCM. COS, core outcome set; HICH, hypertensive intracerebral hemorrhage; TCM, traditional Chinese medicine

A flowchart of the research is shown in Fig. 1.

#### Phase 1: developing a long list of general outcomes

We will develop a long list of general outcomes by making a systematic review and semi-structured interviews.

##### Step 1: systematic review

(i) Search strategy

Eight online databases, Embase, Cochrane Library, PubMed, Web of Science, Wanfang Data, CNKI, VIP, and SinoMed will be searched for the related randomized controlled trials (RCT). We will also search for the China Clinical Trial Registration Center and ClinicalTrials.gov. The literature search will collect relevant information from publications from 1 January 2018 to 1 October 2022.

(ii) Inclusion criteria and exclusion criteria for the literature

The inclusion and exclusion criteria for the literature are shown in Table 1.

**Table 1:** The inclusion and exclusion criteria of systematic review for reported outcomes

Inclusion criteria	Exclusion criteria
Adult patients with HICH	Other causes of intracerebral hemorrhage, for example, aneurysm rupture, blood disease
TCM therapies should be included in the experimental group	Data could not be extracted or there is a serious error
Randomized controlled trials	Full-text cannot be obtained
The clinical trials were published in Chinese or English	Single page, single author published in non-core journal, published by community staff

*HICH *hypertensive intracerebral hemorrhage, *TCM *traditional Chinese medicine.

(iii) Data extraction

Excel was used to set up a data extraction table to extract data. We will mainly extract the following information: the first author’s name, western medicine diagnostic criteria, TCM syndrome names and diagnostic criteria, intervention of experimental group and control group, outcomes, and the methodological quality assessment. All reported outcomes, no matter primary or secondary outcomes, will be listed. At the same time, the definitions, measurement methods and measurement time of outcomes will be recorded, as the authors of the original study report it. The Cochrane handbook will be used to assess the methodological quality [18].

All of the data extraction will be done by two researchers independently. In case of disagreement, it will be resolved by discussion or by seeking the help of a third researcher.

##### Step 2: semi-structured interview

As the list of outcomes generated by systematic review only reflects outcomes considered important by researchers. To take the wishes of patients and other stakeholders into account, patients with HICH and clinicians will be recruited to participate in semi-structured interviews.

(i) Participant selection

The inclusion criteria and exclusion criteria of clinicians and patients for semi-structured interviews are in Tables 2 and 3.

(ii) Sampling strategy

**Table 2:** Inclusion and exclusion criteria of clinicians for semi-structured interviews

Inclusion criteria	Exclusion criteria
Clinicians who have a bachelor’s degree or above	None
Clinicians who have more than 5 years of work experience and senior professional title	
Work in the department of neurology, neurosurgery, or general department

**Table 3:** Inclusion and exclusion criteria of patients for semi-structured interviews

Inclusion criteria	Exclusion criteria
Adult patients with HICH	Patients with severe mental disease
Patients who sign the informed consent forms	
Patients who have the ability to read, understand and speak Chinese	

*HICH* hypertensive intracerebral hemorrhage.

To make sure the views of patients with different experiences and backgrounds are reflected, for example, different age, gender, disease status, and treatment history, we will select specific criteria to provide maximum variation. Heterogeneous, purposeful sampling will be used to recruit patients with HICH in Xiyuan Hospital, China Academy of Chinese Medical Sciences.

(iii) Participant recruitment and data collection

Saturation has attained widespread acceptance as a methodological principle in qualitative research. It is commonly taken to indicate that, on the basis of the data that have been collected or analyzed hitherto, further data collection and/or analysis are unnecessary [19]. This principle will be used in our semi-structured interview, and participants will be no longer included when the information reaches saturation. In addition, some studies have shown that when the sample size was 30, it could achieve data saturation [20, 21]. Therefore, we intended to include 30 clinicians (10 western medicine clinicians and 20 TCM clinicians) at least in 8 hospitals, and 30 HICH patients in our hospital, respectively. However, if new information is generated in the final interview, the sample size of the interview will be expanded. An investigator who is trained in qualitative research methods will be responsible for the semi-structured interviews. First, we will explain the study to the patients. Then, an informed consent form will be signed if the patients agree to participate in the interview. After that, we will begin conducting a semi-structured interview and making an audio recording.

The outline of the semi-structured interviews for clinicians is as follows:

1. How long have you been a clinician?
2. What treatments would you give to a patient with HICH?
3. What outcomes do you think the treatments can improve for the patient?
4. Please write down no more than 5 outcomes that you think are important.

The outline of the semi-structured interviews for patients is as follows:

1. How long have you been diagnosed with HICH?
2. After being diagnosed with HICH, what kind of discomfort did you have?
3. What therapies have you received because of HICH from your doctors?
4. What outcomes do you want to improve after treatment?
5. What outcome do you want the treatment to improve most?

(iv) Data analysis

All the audio recordings of participant interviews will be transcribed verbatim. We will use the framework analysis method to analyze the data. The framework analysis method contains five steps, that are familiarization, identifying a thematic framework, indexing, charting, mapping and interpreting [22]. Two researchers will be responsible for data analysis. In case of disagreement, it will be resolved by discussion or by seeking the help of a third researcher. After that, we will identify outcomes that are important to patients and clinicians respectively.

##### Step 3: merging outcomes and grouping under outcome domains

After the above two tasks are completed, two researchers will be responsible for the consolidation and classification of the outcomes independently. The methods are as follows:

1. Import the extracted outcomes into an Excel table for sorting. The outcomes are numbered and the corresponding research number is matched to facilitate tracing.
2. Remove the duplicate outcomes, record all research numbers and quantities that report the outcomes, and record the use frequency of each outcome.
3. Standardized the extracted original outcomes. For example, abbreviations and nicknames will be standardized, and composite outcomes will be extracted as multiple individual outcomes. And the overlapping outcomes will be merged into one, guaranteeing that the original intention remains unchanged.
4. Obtain the names and frequencies of all outcomes through the first three steps.
5. We will group the outcomes into different outcome domains, according to the taxonomy developed by the COMET initiative [23], also considering the characteristics of TCM.

Finally, two researchers will cross-check the results. In case of disagreement, it will be resolved by discussion or by seeking the help of a third researcher. In order to ensure the process is transparent and clear, we will use the tree diagram.

#### Phase 2: Delphi survey

(i) Stakeholder selection

Healthcare professionals and patients with HICH will be invited to participate in two rounds of the Delphi survey. Health professionals will include TCM clinicians in cerebrovascular disease, Western medicine clinicians in cerebrovascular disease (neurologists and neurosurgeons), researchers interested in HICH, and methodologists in the field of evidence-based medicine.

The inclusion and exclusion criteria for healthcare professionals who will participate in the Delphi survey are as follows:

Inclusion criteria:

1. Health professionals have a bachelor’s degree or above.
2. Health professionals should have more than 1 year of work experience.
3. Clinicians should work in tertiary hospitals.
4. Try to ensure that health professionals come from different regions.
5. The researchers should publish at least one clinical study on cerebrovascular disease.

Exclusion criteria: None.

The inclusion and exclusion criteria for patients who will participate in the Delphi survey are as follows:

Inclusion criteria:

1. Patients with HICH, no restriction on the status.
2. Patients≥18 years old.
3. Patients who have the ability to read, understand, write and speak Chinese.
4. Patients who have signed the informed consent forms.

Exclusion criteria: patients with severe mental disease.

(ii) Sampling strategy

As for the Delphi survey, there is no standard sample size calculation method [24], previous studies ranged from 12 to 174, so we will select 100 stakeholders, including 35 TCM clinicians, 20 western clinicians, 30 patients, 15 researchers and 15 methodologists. The 55 clinicians are all cerebrovascular disease professionals. Because we think outcomes should better reflect the wishes of clinicians and patients, we give them more weight. We will run two rounds of the Delphi survey and finish them in two months. The number of patients recruited will also be based on the information saturation principle, as described in the semi-structured interview.

(iii) Round 1 of the Delphi survey

- Developing a questionnaire for round 1 of the Delphi survey

We will develop a questionnaire for core outcomes according to the list of outcomes obtained by systematic review and semi-structured interviews. If the number of outcomes in the list is more than 80, our questionnaire only includes the top 80 outcomes ranked by frequency according to the previous statistics. If the number of outcomes is not large, all of them can be included in the questionnaire. In round 1 of the Delphi survey, the questionnaire consists of three parts: participants’ basic personal information, the score of the importance of outcomes, and one open-ended question. Participants will use the 9-point Likert Scale [25], advocated by the Grading of Recommendations Assessment, Development and Evaluation working group, to score the importance of all the outcomes in the questionnaire.

Score 1–3 means the outcome is “non-essential,” 4–6 means the outcome is “important but not critical,” and 7–9 means the outcome is “critically important for inclusion.” In addition, considering that participants may lack the expertise to evaluate specific outcomes, we provide an “unclear” option for them to choose. The open-ended question will be placed at the end of the questionnaire for round 1 of the Delphi survey, to know if there are some outcomes the participants think are important but not included in the questionnaire. If so, participants are required to write down the outcomes. For patients, in order to ensure they can better understand the questionnaire, we will write the questionnaire in clear language.

(b) Process of round 1 of the Delphi survey

The survey will last for 2 weeks.

The encephalopathy project team of the China Evidence-based Medicine Center of Traditional Chinese Medicine is composed of 15 tertiary hospitals, which are distributed in 15 provinces. Xiyuan Hospital, China Academy of Chinese Medical Sciences, is the group leader unit of the project team. We will seek the help of these hospitals and ask them to provide a list of health professionals who meet the requirements and volunteer to participate in the Delphi study. For health professionals, a questionnaire and outline of the project will be sent to them by email, to invite them to attend the Delphi survey and complete it within 2 weeks. We will send reminders to the participants who have not completed the survey at 1 week and 48 h remaining for completion of the survey.

We will recruit eligible patients at the inpatient ward or outpatient department of Xiyuan Hospital, China Academy of Chinese Medical Sciences. First, we will give the patient a general overview of our project, and then ask if they want to participate in it. If the patients agree to participate in the two rounds of the Delphi survey, they will sign an informed consent form. After that, our researchers will give a questionnaire to the patient and ask her/him to complete it on the spot.

If the patient has any questions, we will answer them, but we can't induce them to score according to our opinions. At the end of the questionnaire, we will inform the patient to leave his/her contact information, to inform him/her of the time to participate in round 2 of the Delphi survey.

(iii) Data analysis for round 1 of the Delphi survey

We will analyze the results of the health professionals and patients separately. Data analysis for round 1 of the Delphi survey will mainly include: the response rate, the number of replies and score distribution for each outcome, and new outcomes added by participants. As long as more than 10% of participants consider the outcomes important (scored≥4), it will be included in the questionnaire of round 2. For new outcomes, if the steer group believes that the new outcomes are not repeated with the existing outcomes in the questionnaire, the new outcomes will be included in the questionnaire of round 2.

(iv) Round 2 of the Delphi survey

- Developing a questionnaire for round 2 of the Delphi survey

In round 2 of the Delphi questionnaire, we will provide the participants with their own score and score distribution of their own stakeholders from the analysis of round 1 of the Delphi questionnaire. The participants will have the opportunity to reconsider their own scores on these outcomes, using the 9-point Likert Scale. But it is important to point out, if the participant’ score changes too much, such as changing their scores from “not critical” to “critical” or from “critical” to “not critical,” they will be asked to indicate the reasons for the change. Like the round 1 of the Delphi survey, round 2 of the Delphi survey will last for 2 weeks.

For health professionals, a questionnaire and outline of the project will be sent to them by email, to invite them to attend the Delphi round 2 and complete it within 2 weeks. We will send reminders to the participants who have not completed the survey at 1 week and 48 h remaining for completion of the survey. For patients, we will invite them to Xiyuan Hospital, China Academy of Chinese Medical Sciences, to complete the Delphi questionnaire of round 2.

(b) Data analysis for round 2 of the Delphi survey

Data analysis for round 2 of the Delphi survey will mainly include: the response rate, the number of replies, and score distribution for each outcome. According to the results of this round of the Delphi survey, combined with the definitions of a consensus (Table 4), we will preliminarily determine the classification of consensus for each outcome. The outcomes categorized as “consensus out” (Table 4) by all of the stakeholder groups will be excluded. The outcomes categorized as ‘consensus in’ (Table 4) and “no consensus” (Table 4) will be discussed in the consensus meeting.

(iii) Missing data and attrition

**Table 4:** Definitions of a consensus

Classification of consensus	Description	Definition
Consensus in	Consensus that the outcome should be included in the COS.	70% or more participants score the outcome as7 to 9, and <15% of participants score the outcome as 1 to 3 in both stakeholder groups.
Consensus out	Consensus that the outcome should not be included in the COS.	50% or less participants score the outcome as 7 to 9 in both stakeholder group
No consensus	Uncertainty of the importance of outcome.	Anything else.

*COS* core outcome set

Non-response (attrition) and partial response are the two main sources of missing data in the two rounds of the Delphi survey. To reduce non-response, we will send reminders to the participants who have not completed the survey at 1 week and 48 h remaining for completion of the survey, as previously mentioned. To address partial response, only if the participants complete all the Delphi questionnaires of round 1, can they be invited to participate in round 2 of the Delphi survey, and this stipulation will be made clear in the questionnaire of round 1. To evaluate if there is attrition bias, we will calculate the average score of each outcome scored by the participants who complete both rounds or complete round 1 only.

#### Phase 3: consensus meeting

(i) Stakeholder selection

We will conduct a consensus meeting after the completion of the two-round Delphi survey. We will make sure different stakeholders will be invited to attend the consensus meeting, and every group will be represented. At the same time, in order to enhance the recognition and authority of the COS, we will give priority to inviting senior clinical experts in the field of TCM, especially academicians, TCM masters, national famous Chinese medicine practitioners, and academic leaders in this field, no matter if they participate in the Delphi survey. For patients, only patients who have completed two rounds of Delphi surveys will be invited to the consensus meeting.

The inclusion and exclusion criteria for health professionals in the consensus meeting are as follows:

Inclusion criteria:

1. Health professionals with a master’s degree or above.
2. Health professionals who have more than 10 years of work experience.
3. Clinicians who have work experience in tertiary hospitals, and at least have the title of the associate chief physician.
4. Health professionals need to come from different regions.

Exclusion criteria: None.

(ii) Sampling strategy

There is no standard sample size calculation method for the process of the consensus meeting. We will invite 25 participants to attend the consensus meeting, including 9 TCM experts, 5 western medicine experts, 3 researchers, 5 methodological experts and 3 patients.

(iii) Consensus meeting process

We will conduct a face-to-face consensus meeting to determine the final COS after the completion of the two-round Delphi surveys. But if we encounter special circumstances, we will hold a network video conference. It will last at least 1 day.

First, we will report the previous work to the participants, including developing a long list of general outcomes by a systematic review and semi-structured interviews, and two-round Delphi surveys. In particular, we will focus on reporting the results of the second round of the Delphi survey. The outcomes categorized as “consensus in” (Table 4) by all of the stakeholder groups in the second round of the Delphi survey will be preferred to be included in the final COS. The outcomes deemed as “no consensus” (Table 4) will be mainly discussed in the consensus meeting. After discussion, all the participants will use the 9-point Likert Scale to score the importance of the outcomes deemed as “no consensus.” The criteria for consensus used in the Delphi survey (Table 4) will be used at the meeting. After two rounds of the score, if some outcomes are still deemed as “no consensus,” the steering group will decide their classification of consensus. All outcomes categorized as ‘consensus in’ will be included in the final COS. All outcomes deemed as “consensus out” or “no consensus” will not be included in the COS.

After developing the final COS, when and how to measure the included outcomes will be determined. First, we will report the results of measurement time and measurement method, according to the systematic review, and form a questionnaire. Then, participants will discuss it and vote on it. For each outcome in the COS, the measurement time and measurement method with the highest proportion will be recommended at the consensus meeting.

## Patient and public involvement

Patients with HICH will be recruited to participate in semi-structured interviews, two rounds of Delphi survey, and a consensus meeting. At the same time, patients will be involved in the recruitment stage of semi-structured interviews and a Delphi survey. They will be required to recommend relevant known contacts for the study. The purpose of their involvement is to make sure the development of COS can take their wishes into account.

## Dissemination and implementation

After the completion of the final COS, we intend to publish this research in an international journal. And to make sure the report of COS is complete and transparent, we will report it using the Core Outcome Set–STAndards for Reporting (COS-STAR) [23, 26] which consists of a checklist of 18 items.

We will provide publications and a plain summary of results to all the participants by email, so that they can make full use of it in future research. At the same time, so that more health professionals know and use the research results, we will make a special report at the national and international conferences. Also, our findings will be placed on the website of the China Information Association for traditional Chinese medicine and the Pharmacy Clinical Research Information Association (http://www.cria-cm.net).

## Conclusion

Currently, there is no published COS for HICH in clinical trials of traditional Chinese medicine. By developing the COS, we can increase the reporting consistency and reduce the reporting bias in the outcome, which leads to the reuse of research data in meta-analysis and the making of informed healthcare decisions.

## Study status

The study is currently at the systematic literature review stage. Current protocol; version 3 dated 4 January 2021.

## Data Availability

All authors will have access to data.

## Abbreviations

ICH: Intracerebral hemorrhage
HICH: Hypertensive intracerebral hemorrhage
RCTs: Randomized controlled trials
TCM: Traditional Chinese medicine
COS: Core outcome set
COS-STAP: Core Outcome Set Standards for Protocol Items
COS-STAP: Core Outcome Set-STAndardised Protocol
COS-STAR: Core Outcome Set–STAndards for Reporting
